# Comparison and Intercalibration of Vegetation Indices from Different Sensors for Monitoring Above-Ground Plant Nitrogen Uptake in Winter Wheat

**DOI:** 10.3390/s130303109

**Published:** 2013-03-05

**Authors:** Xinfeng Yao, Xia Yao, Wenqing Jia, Yongchao Tian, Jun Ni, Weixing Cao, Yan Zhu

**Affiliations:** National Engineering and Technology Center for Information Agriculture, Jiangsu Key Laboratory for Information Agriculture, Nanjing Agricultural University, Nanjing 210095, China; E-Mails: xinfengyao@saas.sh.cn (X.Y.); yaoxia@njau.edu.cn (X.Y.); 2010101027@njau.edu.cn (W.J.); yctian@njau.edu.cn (Y.T.); nijun@njau.edu.cn (J.N.); caow@njau.edu.cn (W.C.)

**Keywords:** sensors, spectral reflectance, vegetation index, above-ground plant nitrogen uptake, estimating model, comparison, intercalibration, winter wheat

## Abstract

Various sensors have been used to obtain the canopy spectral reflectance for monitoring above-ground plant nitrogen (N) uptake in winter wheat. Comparison and intercalibration of spectral reflectance and vegetation indices derived from different sensors are important for multi-sensor data fusion and utilization. In this study, the spectral reflectance and its derived vegetation indices from three ground-based sensors (ASD Field Spec Pro spectrometer, CropScan MSR 16 and GreenSeeker RT 100) in six winter wheat field experiments were compared. Then, the best sensor (ASD) and its normalized difference vegetation index (NDVI (807, 736)) for estimating above-ground plant N uptake were determined (R^2^ of 0.885 and RMSE of 1.440 g·N·m^−2^ for model calibration). In order to better utilize the spectral reflectance from the three sensors, intercalibration models for vegetation indices based on different sensors were developed. The results indicated that the vegetation indices from different sensors could be intercalibrated, which should promote application of data fusion and make monitoring of above-ground plant N uptake more precise and accurate.

## 1. Introduction

Above-ground plant nitrogen (N) uptake is a good indicator of plant N status [1]. Real-time and accurate monitoring of spatial and temporal variation of above-ground plant N uptake can help farmers make proper N application decisions and improve grain yield and quality [2,3]. The traditional methods for evaluating above-ground plant N uptake, depending on plant tissue analysis, are labor-intensive, time-consuming and expensive, and cannot characterize the temporal and spatial variability of above-ground plant N uptake over large fields. Recently, remote sensing has been proven to be an effective tool to estimate plant N status in the field [4–6].

A wide range of ground-based sensors, working either passively or actively, has been used to produce vegetation indices (VIs) for monitoring vegetation photosynthetic activities and biophysical properties [4,5]. Passive sensor systems, such as the ASD Field Spec Pro spectrometer (Analytical Spectral Devices, Boulder, CO, USA) and CropScan MSR 16 handheld multispectral radiometer (CropScan, Rochester, MN, USA) use sunlight as the source of light. Active sensors such as the GreenSeeker RT 100 (NTech Industries Inc., Ukiah, CA, USA) and Crop Circle ACS-470 (Holland Scientific Inc., Lincoln, NE, USA) are equipped with light-emitting components providing radiation in specific wavelength regions. Passive sensors are mostly multispectral or hyperspectral, enabling the calculation of numerous VIs, thus making themselves more flexible and applicable [7,8], although passive sensors can only be used under adequate light conditions. Active sensors are limited by their use of several central wavelengths and can thus be used to calculate only a few VIs, but they can be used, independent of solar radiation, in the field, even at night [9,10].

The capability and operability of active and passive sensors in monitoring plant growth status have been compared in previous studies [10–12]. Fitzgerald [11] concluded that active sensors did not perform as well as passive sensors in measuring green cover, but differences in model performance were small. The easy operation of active sensors without radiometric calibration would outweigh the small reduction in correlation or sensitivity in RMSE. Fitzgerald also found that the relationships of the typical, sunlight-based NDVI to biomass or leaf area index were nonlinear, while the relationships of NDVI and especially of SAVI from the active sensor to biomass or leaf area index were much more linear. It was proposed that the active sensors could measure the biomass or leaf area index more robustly [11]. Erdle *et al.* compared several indices obtained from four sensors, including one passive and three active sensors, and found that R_760_/R_730_ was the most powerful and temporally stable index for detecting the plant N status of winter wheat. Hence, the estimations from the passive sensor were slightly more precise than those from the active sensors. They concluded that active sensor was more flexible in terms of timeliness and illumination conditions, but it is bound to a limited number of central wavelengths [10].

The normalized difference vegetation index (NDVI), based on the red and near-infrared (NIR) reflectance difference divided by their sum [13], is one of the most widely used indices for monitoring plant N status. NDVI is also in a good correlation with green leaf cover [14], green leaf biomass [15] and grain yield [16]. It can also be used as an indicator of plant development, and can be input into crop models [17]. All common sensors can provide NDVI, but they are varied in central wavelengths or bandwidths for calculating NDVI [18,19].

To be able to take full advantage of NDVI from different sensors, studies have been conducted to analyze the compatibility of NDVI from new and advanced sensor systems with the existing long-term NDVI time series data [18–21]. It was found that cross-sensor differences of NDVI were dependent on variations in solar radiation [22] and in bidirectional response introduced by the different solar radiations and viewing angles [23]. By calibrating the differences in solar radiations and viewing angles and then by correcting the differences in central wavelengths and bandwidths, near equivalent NDVI between sensors can be achieved [18,19,24]. In most of the above studies, the broadband VIs derived from active and passive sensors were compared, but the narrowband VIs constructed by passive sensor were not systematically compared with broadband VIs obtained by both active and passive sensors, and the intercalibration model between the active and passive sensors has been rarely reported.

In precision agriculture, it is necessary to fuse data from more than one sensor to characterize the temporal and spatial variability of above-ground plant N uptake over large fields. On the basis of six field experiments in winter wheat, this study obtained the canopy spectral reflectance from three different ground-based sensors (involving two passive sensors—ASD and CropScan—and one active sensor—GreenSeeker) and above-ground plant N uptake. The main objectives of this study were to: (1) compare the spectral reflectance and VIs derived from different sensors, (2) compare and determine the best sensor and its VI for estimating above-ground plant N uptake, and establish the monitoring model for above-ground plant N uptake in winter wheat; and (3) intercalibrate VIs from different sensors.

## 2. Materials and Methods

### 2.1. Experiment Design

The data included in this study were obtained from six experiments in winter wheat carried out over different years and eco-sites. Treatments under study included different N rates (Exp. 1 and 3), varieties and N rates (Exp. 2 and 4), sowing dates (Exp. 5), and plant densities (Exp. 6), as detailed in Table 1.

### 2.2. Data Measurements

#### 2.2.1. Measurements of Canopy Spectral Reflectance

Three sensors were used to measure canopy spectral reflectance in this paper: (1) ASD Field Spec Pro spectrometer (Analytical Spectral Devices, Boulder, CO, USA), abbreviated as ASD; (2) CropScan MSR 16 handheld multispectral radiometer (CropScan, Rochester, MN, USA), abbreviated as CS; (3) GreenSeeker RT 100 (NTech Industries, Ukiah, CA, USA), abbreviated as GS.

The ASD recorded reflectance between 350 and 1,000 nm, with a sampling interval of 1.4 nm and a resolution of 3 nm, and reflectance between 1,000 and 2,500 nm with a sampling interval of 2 nm and a resolution of 10 nm. It had a 25° field of view fiber optics and was operated at nadir 1.2 m above the winter wheat canopy. The reflected radiance was converted to spectral reflectance by normalization with radiance measured over a white Spectralon reflectance panel (Labsphere, North Sutton, NH, USA). Fifteen scans were obtained for each plot and averaged to produce final canopy spectral reflectance. Radiance measurement of the Spectralon panel was obtained for every fifteen canopy spectral measurements.

**Figure 1.**
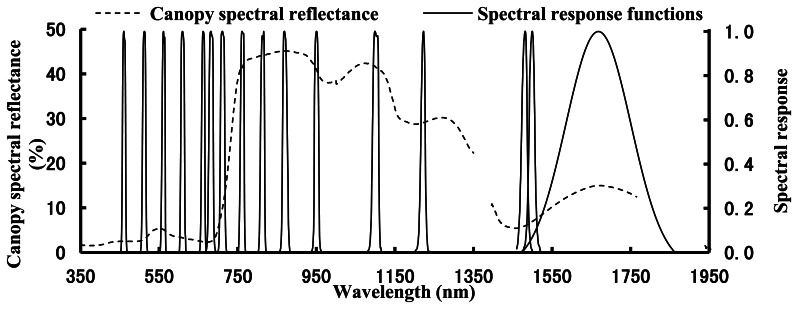
The canopy spectral reflectance of winter wheat and the spectral response function of CS.

The CS measured the canopy reflectance of 16 specific wavebands with each central wavelength and bandwidth between 447 and 1,752 nm (Figure 1). The sensor collected the upwelling radiance of target and downwelling irradiance of solar simultaneously at each central wavelength, using a cosine diffuser for the downwelling irradiance of solar measurement. Thus, it was able to compute reflectance directly rather than through the use of a white Spectralon reference panel and therefore could collect reflectance rapidly. The field of view was 28° and the sensor was operated at nadir 1.2 m above the canopy of winter wheat. Ten scans were obtained for each plot and averaged to produce final canopy spectral reflectance.

The GS used two LEDs as a light source and detected the reflection in the visual (VIS, with 656 nm and 25 nm as the central wavelength and bandwidth, respectively) and near infrared (NIR, with 774 nm and 25 nm as the central wavelength and bandwidth, respectively) spectral regions. The field of view was (24 ± 4)″ × (0.6 ± 0.2)″, and the sensor was operated at nadir 1.2 m above the winter wheat canopy. The sensor was run crosswise according to the sowing direction, and 10 rows of each plot were measured. With reference to the manufacturers' instructions, GS was calibrated before delivery and no additional calibration was further required. In addition, the data obtained from GS was NDVI.

All spectral reference measurements were made under clear sky conditions between 10:00 and 14:00 (Beijing local time). In each experiment, data were obtained on several different dates, which corresponded to the major growth stages, as summarized in Table 1.

#### 2.2.2. Determination of Above-Ground Plant N Uptake

The determination of above-ground plant N uptake was made by the sample of an area of 0.25 m^2^ (two rows and 0.5 m long) of winter wheat plants from each plot. For each sample, the above-ground plant dry weight was measured when the collected plants were oven-dried at 80 °C to constant weight. The above-ground plant total N concentration was determined by the micro-Kjeldahl method. Above-ground plant N uptake (PNU, g·N·m^−2^) was calculated as the product of above-ground plant N concentration on dry weight basis (PNC, g·100·g^−1^) and above-ground plant dry weight per unit ground area (PDW, g·DW·m^−2^).

### 2.3. Data Analysis

#### 2.3.1. Intercalibration and Comparison of Spectral Reflectance between Different Sensors

The central wavelengths of three sensors in this study had different bandwidths; so the spectral response function was used to resample spectral reflectance among different sensors for further comparing and intercalibration. On the basis of spectral response function at 16 central wavelengths of the CS (Figure 1); the spectral reflectance from ASD was resampled; denoted by ASD_CS in this paper. Similarly; the spectral reflectance from ASD was resampled based on the Gauss spectral response function (central wavelengths of 774 nm and 656 nm with bandwidths of 25 nm) of the GS [25]; denoted by ASD_GS.

#### 2.3.2. Calculation of VIs from Different Sensors

The narrowband VIs for estimating above-ground plant N uptake in this paper, as well as their algorithms and sources, were listed in Table 2. Among these indices, NDVI (807, 736) was determined as the optimal index to estimate above-ground plant N uptake based on the all possible two-band combinations of NDVI (λ1, λ2) in our lab [26], and the other VIs were commonly used indices for monitoring plant N status.

Furthermore, the resampled ASD data based on the central wavelengths and bandwidths of CS and GS were used to calculate all possible two-band combinations of NDVI (λ1, λ2) [5], and were abbreviated as NDVI_ASD_CS and NDVI_ASD_GS, respectively. The NDVI based on the original ASD data corresponding to the central wavelengths of CS and GS was abbreviated as NDVI_ASD, and the NDVIs based on the original CS and GS data were abbreviated as NDVI_CS and NDVI_GS, respectively.

#### 2.3.3. Model Calibration

On the basis of calibration data (marked as C in Table 1), the VIs from different sensors in this paper were used to establish linear models for monitoring above-ground plant N uptake in winter wheat. The coefficient of determination (R^2^) and root mean square error (RMSE) were computed to evaluate the performance of the models [33]. Furthermore, the scatter plots between above-ground plant N uptake and VIs were drawn to show the fitness of the models.

#### 2.3.4. Model Validation

The independent data (marked as V in Table 1) were used to test the monitoring models with R^2^, RMSE, slope and intercept of linear equation. The model performance was more accurate with the R^2^ and slope near to 1, while the RMSE and intercept near to 0 [33]. Besides, the 1:1 scatter plots of the predicted and observed values were drawn to display the performance of the models.

Finally, evaluation of VI performance was carried out based on considering both calibration and validation results. In this way, the model's predictive performance was assessed by ranking R^2^ values in decreasing order and RMSE values in ascending order for calibration and validation datasets respectively, while ranking absolute values of (slope-1) and absolute values of intercept in ascending order only for the validation datasets. The overall performance of VIs across calibration and validation datasets was evaluated according to the sum of above six ranks, and the VI with the lowest sum of above six ranks was selected as the best VI [26]. All of the above procedures were implemented using MATLAB 7.9.

## 3. Results

### 3.1. Canopy Spectral Reflectance from Different Sensors at Different Above-Ground Plant N Uptake Levels

**Figure 2.**
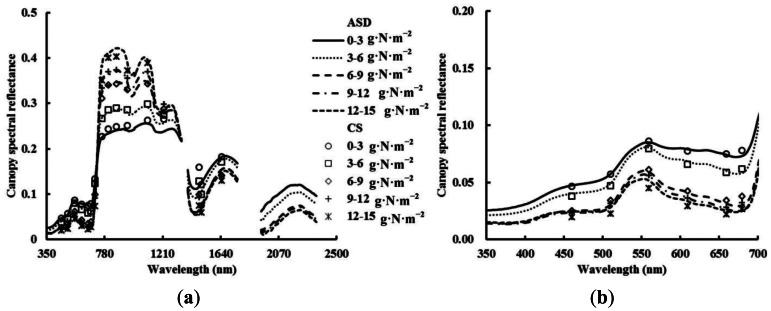
Changes in canopy spectral reflectance from ASD and CS at different above-ground plant N uptake levels in winter wheat. (**a**) 350–2,500 nm; (**b**) 350–700 nm.

Figure 2 shows an identical pattern of the changes in canopy spectral reflectance obtained from ASD and CS at different above-ground plant N uptake levels. In the visible region, the reflectance was in a downward trend with increasing above-ground plant N uptake, and the trend slowed down when the above-ground plant N uptake was above 6–9 g·N·m^−2^. Especially, the reflectance of the red ranges tends to be saturated at relatively high above-ground plant N uptake (Figure 2(b)). However, the reflectance in the NIR region was in an upward trend, and the trend maintained till the above-ground plant N uptake of 12–15 g·N·m^−2^.

### 3.2. Comparison of Canopy Spectral Reflectance from Different Sensors

Significant positive correlations were observed between the canopy spectral reflectance of ASD and that of ASD_CS, with the R^2^ greater than 0.999, except when the central wavelength was at 1,650 nm (figure not shown). The wider bandwidth (195 nm) at the central wavelength of 1,650 nm affected the resampled data (ASD_CS) by atmospheric water absorption wavelengths between the 1,352 nm and 1,451 nm, which significantly reduced the R^2^ (0.321). Significant positive correlations were also observed between the canopy spectral reflectance of ASD and that of ASD_GS, with the R^2^ greater than 0.999 (figure not shown).

**Figure 3.**
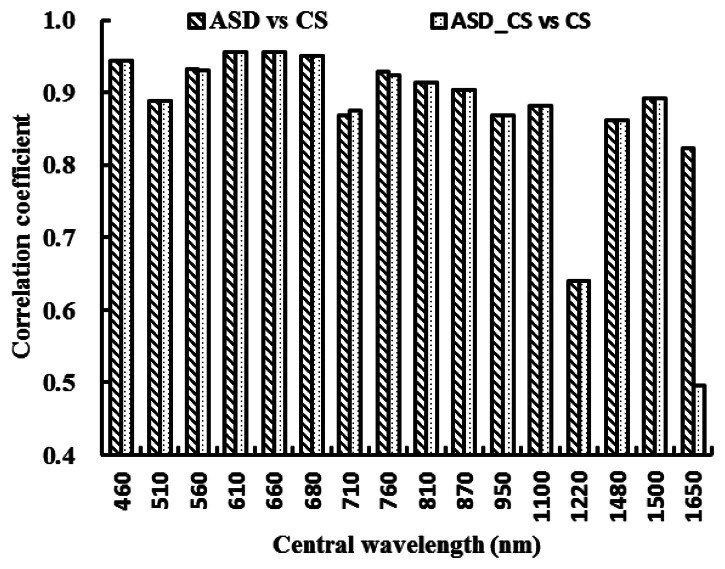
The correlation coefficients of canopy spectral reflectance between ASD and CS, ASD_CS and CS in winter wheat.

Figure 3 shows the correlation coefficients of canopy spectral reflectance between ASD and CS, ASD_CS and CS, respectively. Except at the central wavelengths of 1,220 nm and 1,650 nm, both ASD and ASD_CS reflectance were significantly correlated with the CS reflectance (correlation coefficients were higher than or close to 0.9). At the central wavelength of 1,220 nm, the spectral reflectance of CS were significantly different with that of ASD and ASD_CS, which obviously reduced the correlation coefficients of canopy spectral reflectance between ASD and CS, ASD_CS and CS. At the central wavelength of 1,650 nm, the correlation coefficient of spectral reflectance between ASD and CS was significantly higher than that between ASD_CS and CS, implying that the atmospheric moisture absorption wavelengths could markedly disturb the spectral reflectance of ASD_CS (Figure 3). Figure 3. The correlation coefficients of canopy spectral reflectance between ASD and CS, ASD_CS and CS in winter wheat.

### 3.3. Comparison of VIs among Different Sensors

**Figure 4.**
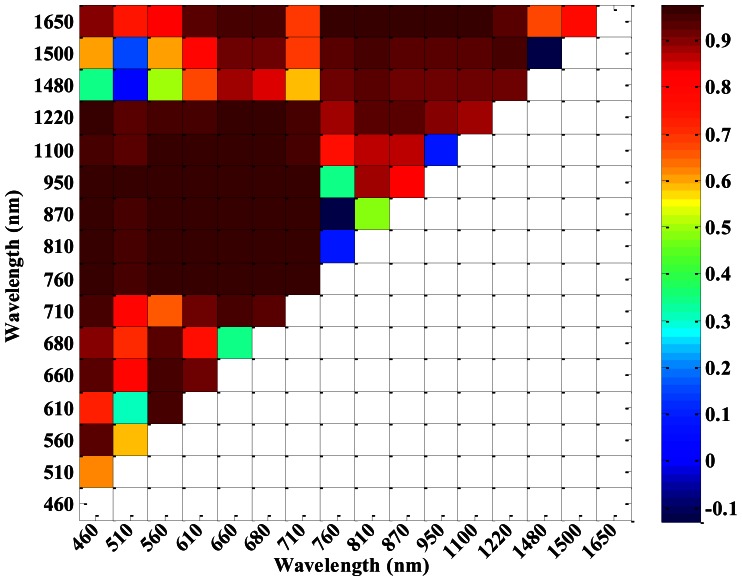
The correlation coefficients of NDVI_CS and NDVI_ASD in winter wheat.

**Figure 5.**
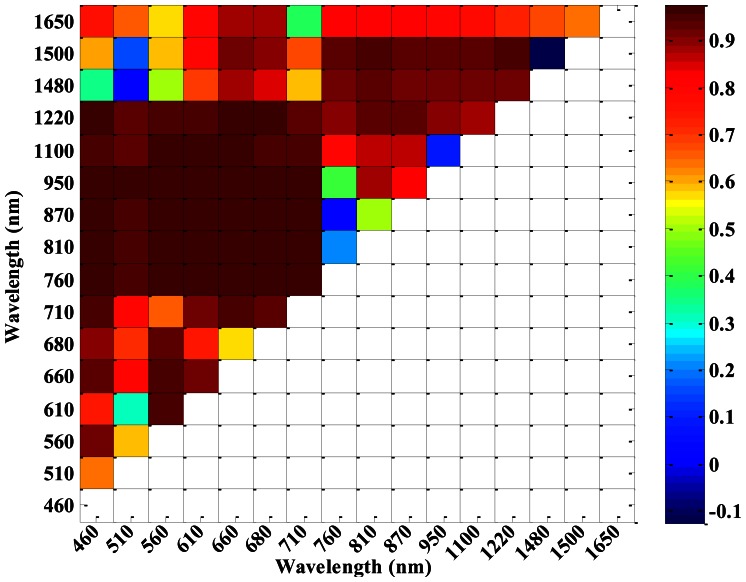
The correlation coefficients of NDVI_CS and NDVI_ASD_CS in winter wheat.

According to the 16 central wavelengths of CS, NDVIs of different sensors (ASD, CS and ASD___CS) with all possible two-band combinations were computed. Figure 4 shows that the correlation coefficients between NDVI_CS and NDVI_ASD varied with different central wavelength combinations. NDVI_CS with central wavelength combinations in the region of 460–710 nm and 760–1,220 nm, as well as 760–1,220 nm and 1,480–1,650 nm, had high correlation coefficients with NDVI_ASD. Meanwhile, the correlation coefficients between NDVI_CS and NDVI_ASD_CS also varied with the different central wavelength combinations (Figure 5), but there were a little difference from those in Figure 4.

**Figure 6.**
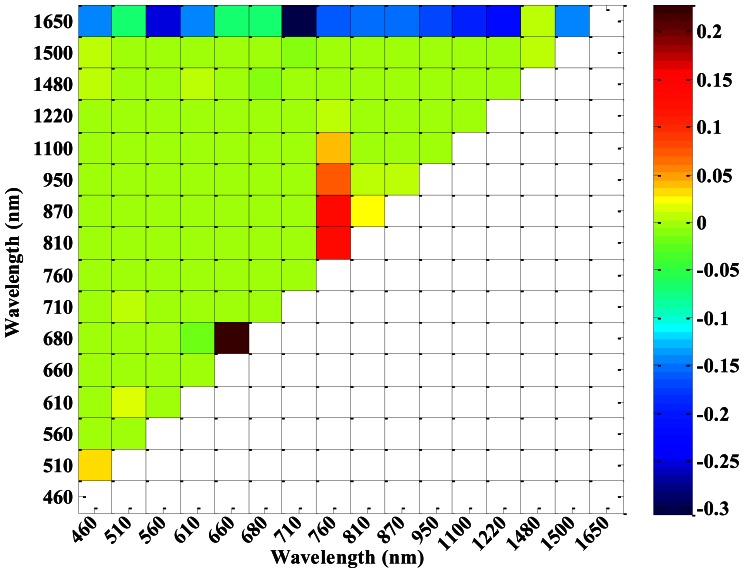
The differences in correlation coefficients of NDVI_CS and NDVI_ASD_CS with NDVI_CS and NDVI_ASD in winter wheat.

In order to compare the correlation coefficients between NDVI_CS and NDVI_ASD_CS and between NDVI_CS and NDVI_ASD, the differences of the correlation coefficients were plotted in Figure 6. Figure 3 shows that the ASD_CS data were similar to the CS data as compared with the ASD data, while, Figure 6 shows that most correlation coefficients between NDVI_CS and NDVI_ASD_CS, were less than those between NDVI_CS and NDVI_ASD, except for the NDVI (660, 680), NDVI (760, 810), NDVI (760, 870) and NDVI (760, 950), and the absolute differences in most two-band combinations were less than 0.05.

**Figure 7.**
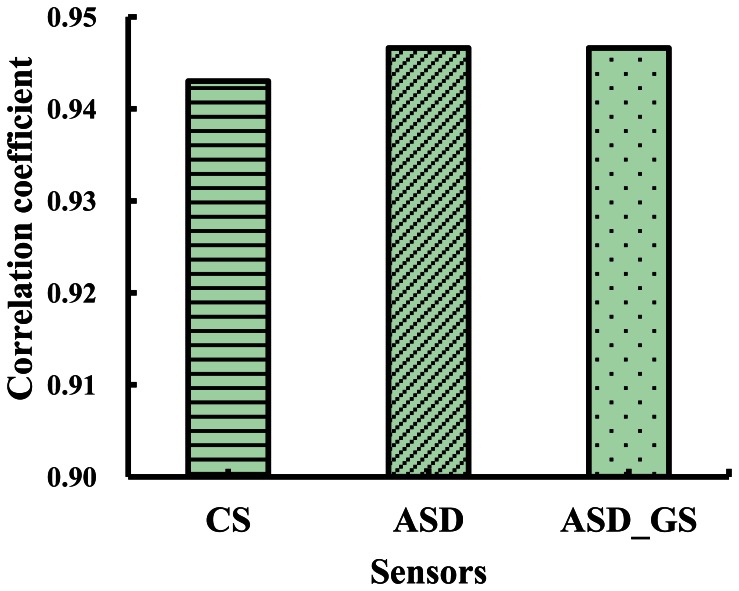
The correlation coefficients between NDVI (774, 656)_GS and NDVI (760, 660)_CS, NDVI (774, 656)_ASD and NDVI (774, 656)_ASD_GS in winter wheat.

Figure 7 shows the correlation coefficients between NDVI (774, 656)_GS and NDVIs from CS, ASD and ASD_GS, including NDVI (760, 660)_CS, NDVI (774, 656)_ASD and NDVI (774, 656)_ASD___GS, in which the central wavelengths in NDVI (760, 660)_CS are the closest central wavelengths of CS compared with GS. There were no obvious differences between NDVI (774, 656)_GS and NDVI (760, 660)_CS, NDVI (774, 656)_GS and NDVI (774, 656)_ASD, and NDVI (774, 656)_GS and NDVI (774, 656)_ASD___GS, respectively (Figure 7). The correlation coefficients between NDVI (774, 656)_GS and NDVI (774, 656)_ASD and between NDVI (774, 656)_GS and NDVI (774, 656)_ASD___GS were relatively higher than those between NDVI (774, 656)_GS and NDVI (760, 660)_CS.

### 3.4. Quantitative Relationships between Above-Ground Plant N Uptake and VIs from Different Sensors

#### 3.4.1. Monitoring Models Based on ASD

**Figure 8.**
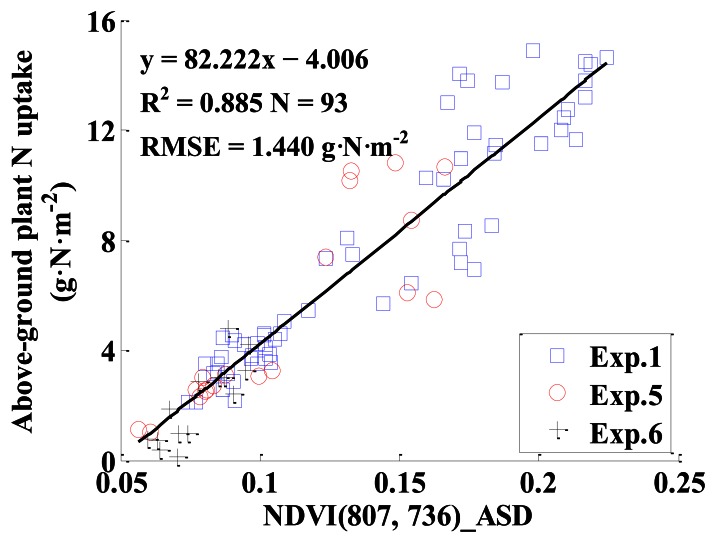
The quantitative relationship between NDVI (807, 736)_ASD and above-ground plant N uptake in winter wheat.

**Figure 9.**
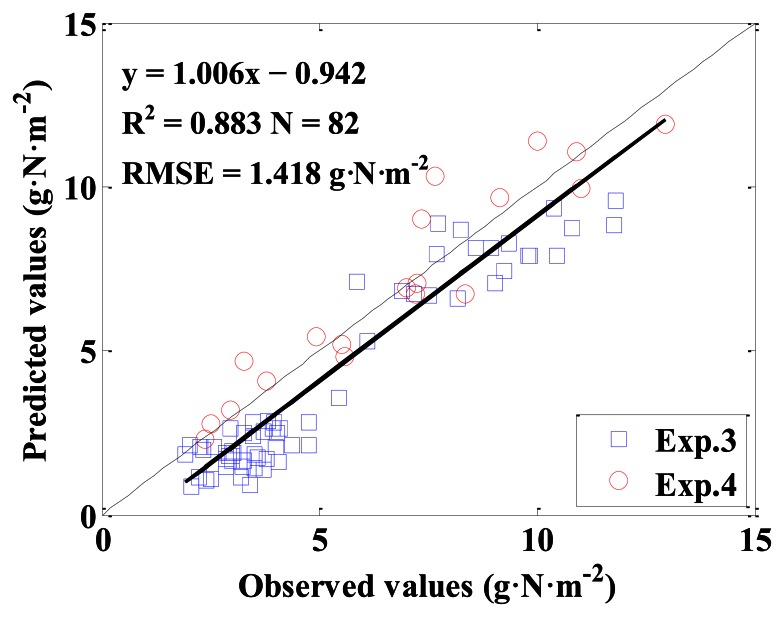
The 1:1 relationship between the predicted and observed above-ground plant N uptake in winter wheat based on NDVI (807, 736)_ASD.

The monitoring models for above-ground plant N uptake with VIs based on ASD in Table 2 were established, and the independent datasets were used to validate the models. Table 3 displays the models and their performance for monitoring above-ground plant N uptake. The results show that the NDVI (807, 736)_ASD was the best VI for monitoring above-ground plant N uptake, with R^2^ of 0.885 and RMSE of 1.440 g·N·m^−2^ for model calibration, and R^2^ of 0.883, RMSE of 1.418 g·N·m^−2^, slope of 1.006 and intercept of −0.942 for model validation. For all VIs, the indices based on the red edge range performed better than those based on the green or red range, as seen in Table 3. Figure 8 shows the relationship between NDVI (807, 736)_ASD and above-ground plant N uptake. Figure 9 displays the 1:1 relationship between the predicted and observed above-ground plant N uptake based on the monitoring model with NDVI (807, 736)_ASD.

#### 3.4.2. Monitoring Models Based on CS

**Figure 10.**
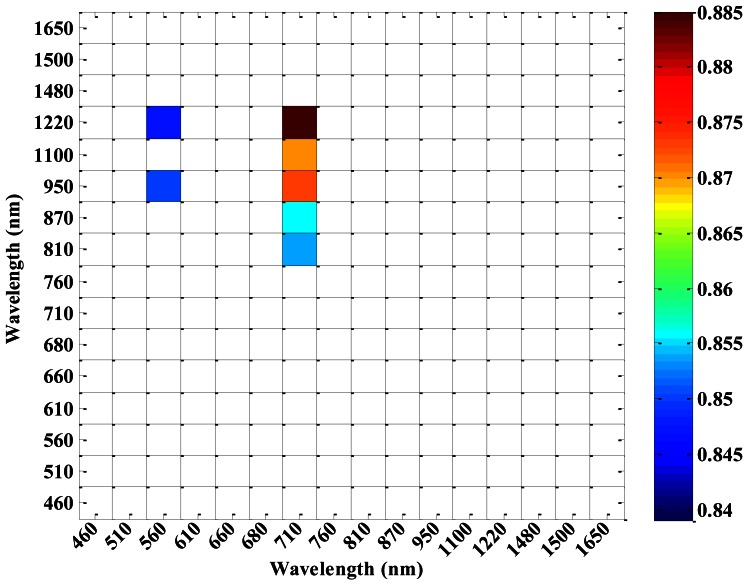
The top 5% R^2^ of monitoring models for above-ground plant N uptake in winter wheat based on NDVIs from CS.

NDVI_CS with all possible two-band combinations of NDVI (λ_1_, λ_2_) were computed and the monitoring models between NDVI_CS and above-ground plant N uptake were established. Figure 10 shows the top 5% R^2^ of all the monitoring models for above-ground plant N uptake based on NDVI_CS. The statistics parameters were listed in Table 4. Among all the NDVI_CS, the best index for estimating above-ground plant N uptake was NDVI (950, 710)_CS. The model performance was good with R^2^ of 0.873, RMSE of 1.514 g·N·m^−2^, for calibration, and with R^2^ of 0.856, RMSE of 1.393 g·N·m^−2^, slope of 0.936 and intercept of −0.425 for validation.

**Figure 11.**
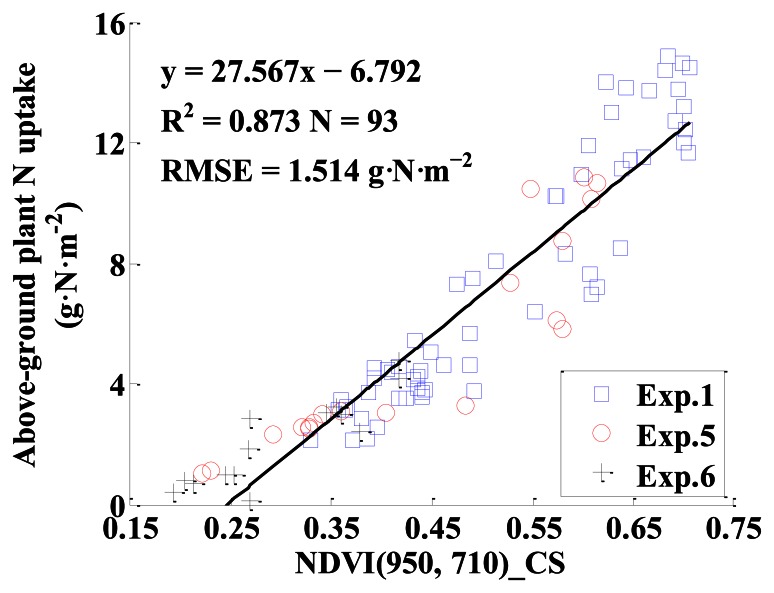
The quantitative relationship between NDVI (950, 710)_CS and above-ground plant N uptake in winter wheat.

**Figure 12.**
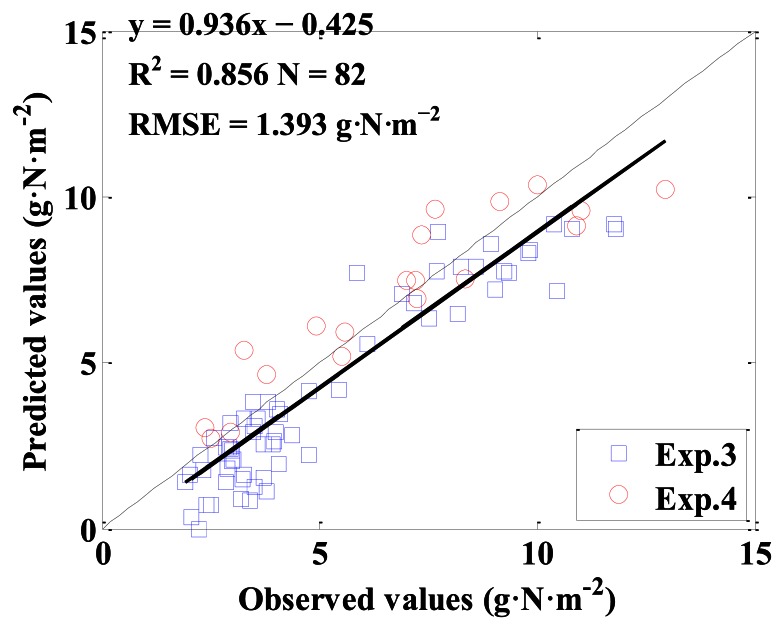
The 1:1 relationship between the predicted and observed above-ground plant N uptake based on NDVI (950, 710)_CS.

Figure 11 shows the linear relationship between NDVI (950, 710)_CS and above-ground plant N uptake. Figure 12 shows a 1:1 relationship between the predicted and observed above-ground plant N uptake based on NDVI (950, 710)_CS.

#### 3.4.3. Monitoring Models Based on GS

**Figure 13.**
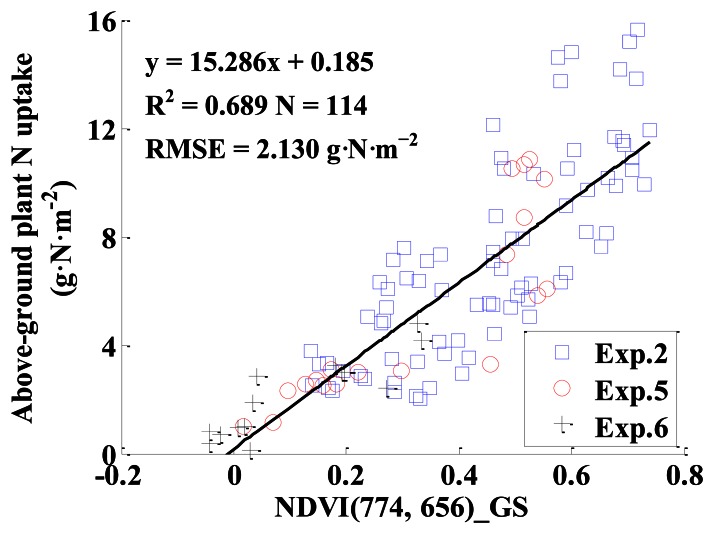
The quantitative relationship between NDVI (774, 656)_GS and above-ground plant N uptake in winter wheat.

**Figure 14.**
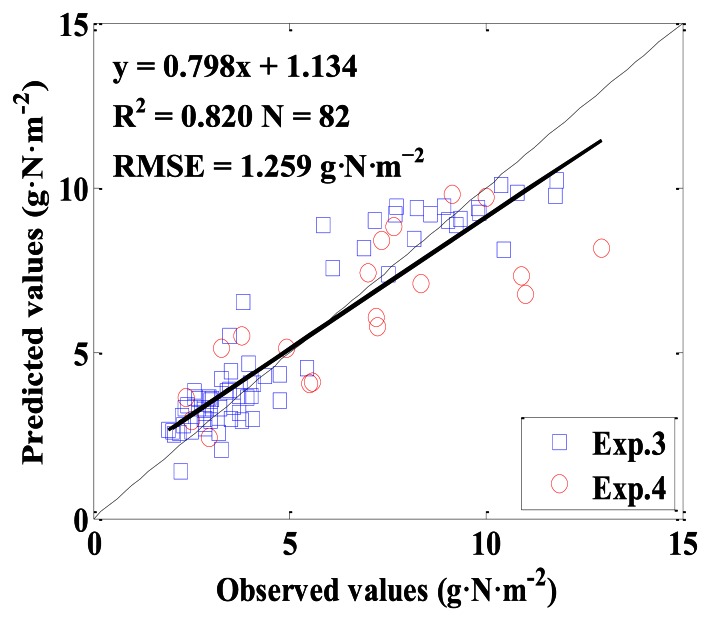
The 1:1 relationship between the predicted and observed above-ground plant N uptake based on NDVI (774, 656)_GS in winter wheat.

NDVI (774, 656)_GS was used to develop a linear model for estimating above-ground plant N uptake, and the model was y = 15.286x + 0.185, with R^2^ and RMSE being 0.689 and 2.130 g·N·m^−2^ for calibration, and with R^2^, RMSE, slope and intercept being 0.820, 1.259 g·N·m^−2^, 0.798 and 1.134 for validation, respectively. The model performance for calibration was not very satisfactory, because the linear relationship between NDVI (774, 656)_GS and above-ground plant N uptake did not exist when the above-ground plant N uptake was higher than 10 g·N·m^−2^ (Figure 13). The result of model validation was consistent with that of calibration,when the above-ground plant N uptake was lower than 10 g·N·m^−2^, the predictive performance of the model had a 1:1 relationship between the predicted and observed values (Figure 14).

### 3.5. Comparison of VIs from Resampled Reflectance for Estimating Above-Ground Plant N Uptake

Based on the central wavelength combinations with top 5% R^2^ in CS, monitoring models from ASD and ASD_CS data were established (Table 5).

Among all NDVIs_ASD, the NDVI (950, 710)_ASD performed best for estimating above-ground plant N uptake, with R^2^ higher than 0.84 for calibration and R^2^ higher than 0.85 for validation. Among all the NDVIs_ASD_CS, the NDVI (950, 710)_ASD_CS displayed similar results with those of NDVI (950, 710)_ASD for estimating above-ground plant N uptake.

Based on the central wavelengths of GS (774 nm and 656 nm), NDVI (774, 656)_ASD, NDVI (774, 656)_ASD_GS and NDVI (760, 660)_CS (closest central wavelengths of CS compared with GS) were obtained for estimating above-ground plant N uptake in winter wheat. Comparing the performances of the monitoring models based on these VIs, it could be found that NDVI (774, 656)_ASD, NDVI (774, 656)_ASD_GS and NDVI (774, 656)_GS had similar results for estimating above-ground plant N uptake, and their performances were worse than NDVI (760, 660)_CS (Tables 4 and 5, and Figure 13).

It could be concluded that among the models for estimating above-ground plant N uptake based on VIs from ASD, CS, GS, ASD_CS and ASD_GS, the best index for estimating above-ground plant N uptake in winter wheat was NDVI (807, 736)_ASD, with the highest R^2^ of 0.885 and 0.883 for calibration and validation, respectively (Tables 3, 4 and 5 and Figure 13).

### 3.6. Intercalibration of Optimal VI from ASD and CS

The above results indicated that the spectral reflectance of the original ASD (ASD) and the resampled ASD data (ASD_CS, ASD_GS) were not significantly different in most central wavelengths and in the NDVIs. The intercalibration models based on original and resampled data were constructed and compared, and the results showed that there was no significant difference between them (figure not shown). Thus the intercalibration of VIs from different sensors (ASD, CS and GS) was based on the original sensor data in this paper.

Table 6 reports the coefficients of determination (the higher one between the models based on linear and quadratic polynomial) between the VIs from ASD and CS. The result showed that NDVI (807, 736)_ASD and NDVI (810, 710)_CS had the highest coefficient of determination (0.9720) (Table 6). The results of Section 3.4.1 showed that NDVI (807, 736)_ASD was the best index among the NDVIs_ASD for estimating above-ground plant N uptake, while the results of Section 3.4.2 showed that NDVI (950, 710)_CS was the best index among the NDVIs_CS. However, Table 4 indicates that the performance of NDVI (810, 710)_CS was just slightly lower than NDVI (950, 710)_CS, and it is also good. In addition, the central wavelengths in the NDVI (810, 710)_CS were closer to those in the NDVI (807, 736)_ASD than those in the NDVI (950, 710)_CS. These results suggest that the NDVI (807, 736)_ASD and NDVI (810, 710)_CS should be selected for intercalibration as the optimal VI between ASD and CS.

The intercalibration model based on NDVI (807, 736)_ASD and NDVI (810, 710)_CS was developed. The quantitative relationship between NDVI (807, 736)_ASD and NDVI (810, 710)_CS was in the form of a quadratic polynomial model as y = −13.831x^2^ + 6.269x−0.070, with R^2^ of 0.972 and RMSE of 0.021. Furthermore, the model validation results showed that the intercalibration model performed well for the independent data, with R^2^ of 0.953 and RMSE of 0.057.

### 3.7. Intercalibration of NDVI (NIR, red) from ASD, CS and GS

#### 3.7.1. Intercalibration of NDVI (774, 656)_ASD and NDVI (774, 656)_GS

The quantitative relationship between NDVI (774, 656)_ASD and NDVI (774, 656)_GS was in the form of a linear model as *y* = 1.076*x* − 0.362, with R^2^ of 0.896 and RMSE of 0.056. Furthermore, the model validation results showed that the intercalibration model performed well for the independent data, with R^2^ of 0.974 and RMSE of 0.077.

#### 3.7.2. Intercalibration of NDVI (774, 656)_GS and NDVI (760, 660)_CS

The quadratic polynomial model of y = −1.016x^2^ + 1.500x + 0.303 was developed to convert NDVI (774, 656)_GS to NDVI (760, 660)_CS, with R^2^ of 0.927 and RMSE of 0.036. On the basis of the intercalibration model, the transformed NDVI (760, 660)_CS was in a 1:1 relationship with measured NDVI (760, 660)_CS, with R^2^ of 0.974 and RMSE of 0.035.

#### 3.7.3. Intercalibration of NDVI (774, 656)_ASD and NDVI (760, 660)_CS

The relationship between NDVI (774, 656)_ASD and NDVI (760, 660)_CS could be fitted as linear equation of *y* = 0.826*x* + 0.127, with R^2^ of 0.942 and RMSE of 0.032. The independent data for validation indicated that the intercalibration model was reliable, with R^2^ of 0.970 and RMSE of 0.076 between predicted and measured NDVI (760, 660)_CS.

## 4. Discussion

### 4.1. Spectral Reflectance and NDVI among Different Sensors

Because of different design principles for different sensors, some differences could be found in the reflectance of single wavelength and in NDVIs obtained from different sensors at the same central wavelength, even in those of the resampled data (Figures 2, 3, 4, 5, 6 and 7).

Two approaches are often used to make reflectance measurements on the ground-based passive sensor. The first is sequential measurement, in which solar irradiance is measured by periodically taking radiance from a calibration panel with known reflectance; and the reflected radiance of the ground target (generally the crop) is measured between two measurements of calibration panel. Then, the reflectance of the targets is calculated by dividing the radiance of the targets by the radiance of the calibration panel. The second is simultaneous measurement, in which irradiance values from the solar and from the target are measured simultaneously. Reflectance is also calculated by dividing radiance from the target by solar irradiance [34].

In this paper, the sequential measurement was used for the ASD, and the simultaneous measurement was used for the CS to determine the reflectance of the target. Using the ASD to measure the reflectance of the target, the variations could occur in atmospheric transmission between the measurements of the target radiance and solar irradiance, and substantial errors might occur in the calculated reflectance [35]. Previous studies showed that, under the condition of stable illumination, the sequential measurement introduced only slightly more variance in computed reflectance than simultaneous measurements. However, when clouds were present, even not immediately adjacent to the sun, solar irradiance varied over short temporal periods, then significant differences in the reflectance of target occurred between two different measurements [36].

Active sensors do not depend on sunlight or other external light sources, and they can be used under inadequate light conditions, even at night. They do not require a calibration panel because the light source intensity is known and constant. However, since the light source of active sensors has a limited range, they are limited to proximal sensing only. The commercial sensors only provide two central wavelengths, e.g., GreenSeeker RT 100 provides two central wavelengths of 774 nm and 656 nm, from which the normalized difference vegetation index (NDVI), ratio vegetation index (RVI) [13], soil-adjusted vegetation index (SAVI) [32] or related indices could be calculated.

However, during the measurements in the present study, the fields of view of the sensors were different while the same measurement height was used, thus different canopy sizes were measured with different sensors. In future study, the researchers should adjust the height of the sensors to ensure the similar canopy in the fields of view of different sensors.

### 4.2. Monitoring Model for Above-Ground Plant N Uptake among Different Sensors

This paper also compared the quantitative relationships between VIs derived from different sensors and above-ground plant N uptake, from which some interesting results were found. Firstly, according to the measuring principle of sensors, GS should be the most reliable sensor in this paper; however it did not provide the best estimation of above-ground plant N uptake. When the above-ground plant N uptake was higher than 10 g·N·m^−2^, the NDVI (774, 656)_GS was saturated (Figures 13 and 14). The saturation effects of NDVI from GS in current study were consistent with previous studies in maize [9] and wheat [10] crops. With high biomass, above-ground plant N uptake and LAI, the reflectance of red range tended to be saturated sooner than NIR range [37], and the central wavelength in red range was used in GS [10], which caused the saturation.

Secondly, the NDVIs of the same central wavelengths from CS performed better than those from ASD and ASD_CS (Tables 4 and 5). The results indicated that the reflectance of CS were more reliable than the ASD, which might be that the CS measured the target radiance and the solar irradiance simultaneously, and could reduce errors of the change in solar irradiance.

Thirdly, among the VIs from the above three sensors, the narrowband VI of NDVI (807, 736) from ASD performed the best for above-ground plant N uptake estimation (*y* = 82.222*x* − 4.006), with R^2^ of 0.885 and RMSE of 1.440 g·N·m^−2^ for model calibration, and with R^2^ of 0.883, RMSE of 1.418 g·N·m^−2^, slope of 1.006 and intercept of −0.942 for model validation. This finding was consistent with those of Mutanga and Skidmore [37] and Thenkabail *et al.* [4], who found that the NDVIs based on novel narrowbands could overcome the saturation of standard NDVI and could extract biomass information in areas of dense vegetation with a high degree of accuracy. The ASD sensor could provide a huge number of narrowbands to be used to construct new VIs for monitoring crop growth status. These results indicated that central wavelengths were, to a certain extent, more important than measuring principle of sensors in model accuracy.

These findings in the present paper should be helpful for designing passive sensors with simultaneous measurements or active sensors with new central wavelengths (e.g., 807 and 736 nm), which could monitor plant growth status, especially N status, more precisely and accurately.

### 4.3. Intercalibration of Different Sensors

This study suggested that the original (CS and GS) and resampled spectral reflectance (ASD_CS and ASD_GS) had no significant difference in most central wavelengths and in the NDVIs respectively. These findings were different from previous studies in which resampled data, based on sensor specific spectral response functions instead of original data, were used to intercalibrate NDVI (NIR, red) between different sensors in order to reduce the error [18,19]. It might be that the sensors intercalibrated in previous studies were satellite sensors with broader bandwidths, while the sensors used in this paper were ground-based sensors with narrower bandwidths. Therefore, the original data of ASD, CS and GS were used to intercalibrate VIs from different sensors in the present study.

Previous studies indicated that NDVI from different sensors could not be regarded as directly equivalent, because the working principle, central wavelengths and bandwidths were different. However, it could be further found that correlations existed between the NDVIs from different sensors [18,19]. The optimal VI of ASD and CS were intercalibrated based on NDVI (807, 736)_ASD and NDVI (810, 710)_CS. According to the model of y = −13.831*x*^2^ + 6.269*x* − 0.070, NDVI (807, 736)_ASD could be accurately converted to NDVI (810, 710)_CS, with R^2^ of 0.972 and RMSE of 0.021.

There was a strong linear empirical relationship between NDVI (774, 656)_ASD and NDVI (774, 656)_GS, and the two NDVIs could be intercalibrated through *y* = 1.076*x* − 0.362, with R^2^ of 0.896 and RMSE of 0.056 for calibration. The quadratic polynomial empirical relationships were found between NDVI (774, 656)_GS and NDVI (760, 660)_CS, and the two NDVIs could be intercalibrated through *y* = −1.016*x*^2^ − 1.500*x* + 0.303, with R^2^ of 0.927 and RMSE of 0.036 for calibration. There was also a linear relationship between NDVI (774, 656)_ASD and NDVI (760, 660)_CS, and the two NDVIs could be intercalibrated through *y* = 0.826*x* + 0.127, with R^2^ of 0.942 and RMSE of 0.032 for calibration. The above intercalibration models were all validated with independent data, and the results showed that, with these models, nearly equivalent NDVIs between sensors could be achieved. These results were important for monitoring crop growth status with different sensors, and for better continuity of long-term monitoring of vegetation responses to environmental changes.

## 5. Conclusions

In this paper, VIs derived from different ground sensors (ASD Field Spec Pro spectrometer, CropScan MSR 16 and GreenSeeker RT 100) were compared and intercalibrated based on six experiments with winter wheat involving different years, eco-sites, varieties, N rates, sowing dates and sowing densities. The best sensor (ASD) and its VI (NDVI (807, 736)_ASD) were determined to estimate above-ground plant N uptake with higher precision (R^2^ of 0.885 and RMSE of 1.440 g·N·m^−2^). The intercalibration models of VIs between three different sensors were developed. The results would contribute to the application of data fusion and improve the precision and accuracy in monitoring above-ground plant N uptake in winter wheat.

## Figures and Tables

**Table 1.** Main details of the six field experiments.

**Exp. Number**	**Year and Location**	**Variety**	**Sowing Date (Month-Day)**	**Treatment**	**Sampling Date (Month-Day)**	**Sample Number**	**Sensors Comparison**			**Sensors Intercalibration**

							**ASD**	**CS**	**GS**	**ASD & CS & GS**
Exp.1	2007–2008 Nanjing	Ningmai 9	11–5	Nitrogen (kg·hm^−2^): 0, 90, 180, 270	03–11 (early jointing), 03–25 (jointing), 04–18 (heading)	N = 60	C	C		
Exp.2	2009–2010 Yangzhou	Yangmai 16 Ningmai 13	11–3	Nitrogen (kg·hm^−2^): 0, 120, 180, 240, 300	03–28 (jointing), 04–08 (early booting), 04–16 (heading)	N = 81			C	
Exp.3	2009–2010 Yangzhou	Yangmai 16	11–2	Nitrogen (kg·hm^−2^): 0, 90, 180, 270	03–11 (early jointing), 03–19 (jointing), 04–15 (heading)	N = 63	V	V	V	C
Exp.4	2010–2011 Yangzhou	Yangmai 16 Ningmai 13	11–5	Nitrogen (kg·hm^−2^): 0, 75, 150, 225, 300	03–29 (jointing)	N = 19	V	V	V	C
Exp.5	2010–2011 Yangzhou	Yangmai 16	see treatment	Sowing date: 10–15, 10–25, 11–04, 11–14, 11–24	02–24 (green returning)	N = 14	C	C	C	V
Exp.6	2010–2011 Yangzhou	Yangmai 16	11–4	Density (plant·m^−2^) 60, 150, 240, 330, 420	02–23 (green returning), 03–30 (jointing)	N = 19	C	C	C	V

Notes: C was data for model calibration, and V was data for model validation.

**Table 2.** VIs for estimating above-ground plant N uptake in winter wheat.

**VI**	**Algorithm**	**Reference**
RI red edge	(*R_750-800_/R_695-740_*) −1	Gitelson *et al.* (2003) [27]
mSR705	$\frac{R_{750}-R_{445}}{R_{705}-R_{445}}$	Sims and Gamon, (2002) [28]
mND705	$\frac{R_{750}-R_{705}}{R_{750}+R_{705}-2*R_{445}}$	
VOGa	*R*_740_/ *R*_720_	Vogelmann *et al.* (1993) [29]
VOGb	$\frac{R_{734}-R_{747}}{R_{715}+R_{726}}$	
VOGc	$\frac{R_{734}-R_{747}}{R_{715}+R_{720}}$	
PSSRb	*R*_800_/*R*_635_	Blackburn, (1998) [30]
GREEN-NDVI	$\frac{R_{750}-R_{550}}{R_{750}+R_{550}}$	Gitelson *et al.* (1996) [31]
SAVI	$\left(1+0.5\right)\frac{R_{807}-R_{736}}{R_{807}+R_{736}+0.5}$	Huete, (1988) [32]
NDVI (830, 660)	$\frac{R_{830}-R_{660}}{R_{830}+R_{660}}$	Tucker, (1979) [13]
NDVI (807, 736)	$\frac{R_{807}-R_{736}}{R_{807}+R_{736}}$	Yao *et al.* (2012) [26]

**Table 3.** The performance of models for monitoring above-ground plant N uptake in winter wheat based on VIs from ASD.

**VI**	**Calibration**			**Validation**				**Summed Rank**

	**Model**	**R^2^**	**RMSE (g·N·m^−2^)**	**R^2^**	**RMSE (g·N·m^−2^)**	**Slope**	**Intercept**	
NDVI (807,736)	*y* = 82.222*x* − 4.006	0.885	1.440	0.883	1.418	1.006	−0.942	15
VOGb	*y* = −44.012*x* − 0.725	0.878	1.482	0.868	1.560	0.979	−0.952	26
mSR705	*y* = 4.028*x* − 2.340	0.854	1.624	0.858	1.352	1.028	−0.681	28
VOGc	*y* = −36.799*x* − 0.336	0.876	1.496	0.866	1.544	0.956	−0.818	28
PSSRb	*y* = 0.880*x* − 0.251	0.846	1.668	0.842	1.329	0.955	−0.240	32
RI red edge	*y* = 8.714*x* − 2.435	0.876	1.497	0.864	1.551	1.047	−1.191	36
NDVI (830, 660)	*y* = 22.785*x* − 9.858	0.727	2.218	0.788	1.541	0.996	−0.111	40
SAVI	*y* = 15.333*x* − 9.798	0.730	2.209	0.787	1.546	0.997	−0.142	41
VOGa	*y* = 13.346*x* − 15.651	0.864	1.566	0.857	1.718	1.046	−1.403	44
GREEN-NDVI	*y* = 30.882*x* − 12.198	0.792	1.940	0.839	1.533	1.054	−0.964	48
mND705	*y* = 28.628*x* − 10.322	0.801	1.895	0.816	2.258	1.185	−2.439	58

**Table 4.** The performance of models for monitoring above-ground plant N uptake in winter wheat based on NDVIs from CS.

**VI**	**Calibration**			**Validation**				**Summed Rank**

	**Model**	**R^2^**	**RMSE (g·N·m^−2^)**	**R^2^**	**RMSE (g·N·m^−2^)**	**Slope**	**Intercept**	
NDVI (950, 710)	*y* = 27.567*x* − 6.792	0.873	1.514	0.856	1.393	0.936	−0.425	18
NDVI (1100, 710)	*y* = 27.279*x* − 6.974	0.870	1.533	0.857	1.335	0.866	0.294	19
NDVI (870, 710)	*y* = 25.082*x* − 5.798	0.856	1.612	0.850	1.352	0.909	−0.183	20
NDVI (1220, 710)	*y* = 39.074*x* − 11.119	0.885	1.442	0.843	1.576	0.970	−0.798	23
NDVI (950, 560)	*y* = 32.631*x* − 14.138	0.850	1.648	0.832	1.385	0.942	−0.237	26
NDVI (810, 710)	*y* = 24.912*x* − 5.669	0.854	1.621	0.844	1.480	0.933	−0.486	32
NDVI (1220, 560)	*y* = 43.819*x* − 20.236	0.847	1.661	0.826	1.467	0.961	−0.436	34
NDVI (760, 660)	*y* = 21.03*x* − 8.218	0.742	2.158	0.789	1.434	0.813	1.473	45

**Table 5.** The performance of models for monitoring above-ground plant N uptake based on VIs from ASD, ASD_CS and ASD_GS in winter wheat.

**Sensor**	**VI**	**Calibration**			**Validation**				**Summed Rank**

		**Model**	**R^2^**	**RMSE (g·N·m^−2^)**	**R^2^**	**RMSE (g·N·m^−2^)**	**Slope**	**Intercept**	
ASD	NDVI (950, 710)	*y* = 31.436*x* − 7.113	0.849	1.651	0.857	1.634	1.072	−1.369	23
	NDVI (1220, 560)	*y* = 39.039*x* − 16.295	0.779	1.997	0.863	1.289	1.074	−0.394	24
	NDVI (950, 560)	*y* = 31.918*x* − 13.419	0.809	1.858	0.852	1.370	1.017	−0.631	24
	NDVI (1100, 710)	*y* = 32.707*x* − 8.18	0.836	1.723	0.859	1.529	1.119	−1.295	29
	NDVI (1220, 710)	*y* = 42.024*x* − 9.301	0.783	1.981	0.875	1.410	1.161	−1.137	30
	NDVI (870, 710)	*y* = 29.234*x* − 6.684	0.848	1.659	0.852	1.729	1.112	−1.613	31
	NDVI (760, 660)	*y* = 21.719*x* − 9.001	0.718	2.258	0.778	1.561	0.984	0.057	34
	NDVI (774, 656)	*y* = 22.244*x* − 9.387	0.658	2.236	0.801	1.439	0.924	−0.019	36
	NDVI (810, 710)	*y* = 28.768*x* − 6.215	0.845	1.673	0.847	1.786	1.127	−1.717	39
	
ASD_CS	NDVI (950, 710)	*y* = 32.023*x* − 7.266	0.849	1.653	0.858	1.618	1.072	−1.345	21
	NDVI (1220, 560)	*y* = 38.967*x* − 16.304	0.780	1.995	0.863	1.292	1.075	−0.395	22
	NDVI (950, 560)	*y* = 31.887*x* − 13.435	0.808	1.860	0.851	1.370	1.018	−0.627	23
	NDVI (1100, 710)	*y* = 33.299*x* − 8.336	0.835	1.727	0.859	1.516	1.118	−1.259	26
	NDVI (870, 710)	*y* = 29.698*x* − 6.802	0.847	1.660	0.852	1.718	1.111	−1.593	28
	NDVI (1220, 710)	*y* = 43.158*x* − 9.58	0.779	1.997	0.875	1.407	1.163	−1.097	30
	NDVI (760, 660)	*y* = 21.805*x* − 9.094	0.716	2.262	0.779	1.562	0.987	0.000	31
	NDVI (810, 710)	*y* = 29.208*x* − 6.328	0.845	1.675	0.847	1.777	1.126	−1.699	36
	
ASD_GS	NDVI (774, 656)	*y* = 22.243*x* − 9.371	0.656	2.240	0.801	1.432	0.919	0.021	6

**Table 6.** The coefficients of determination between the VIs from ASD and CS.

	**VI from CS**								
	
**VI from ASD**	**NDVI (1220, 710)**	**NDVI (1100, 710)**	**NDVI (950, 710)**	**NDVI (870, 710)**	**NDVI (810, 710)**	**NDVI (950, 560)**	**NDVI (1220, 560)**	**NDVI (760, 660)**	**NDVI (810, 660)**
NDVI (807, 736)	0.9421	0.9694	0.9718	0.9712	0.9720	0.9682	0.9480	0.9263	0.9346
RI red edge	0.9380	0.9642	0.9716	0.9705	0.9710	0.9658	0.9443	0.9348	0.9429
mSR705	0.9242	0.9610	0.9593	0.9597	0.9593	0.9524	0.9275	0.9452	0.9488
mND705	0.9384	0.9564	0.9668	0.9650	0.9660	0.9610	0.9441	0.9225	0.9323
VOGa	0.9352	0.9583	0.9692	0.9679	0.9691	0.9641	0.9430	0.9273	0.9370
VOGb	0.9377	0.9623	0.9716	0.9700	0.9708	0.9654	0.9448	0.9262	0.9349
VOGc	0.9385	0.9625	0.9711	0.9695	0.9702	0.9645	0.9452	0.9253	0.9336
PSSRb	0.9194	0.9522	0.9401	0.9397	0.9378	0.9282	0.9161	0.9272	0.9261
GREEN-NDVI	0.9365	0.9658	0.9613	0.9608	0.9598	0.9549	0.9371	0.9443	0.9487
SAVI	0.9116	0.9472	0.9383	0.9398	0.9388	0.9306	0.9100	0.9435	0.9442
NDVI (830, 660)	0.9119	0.9469	0.9378	0.9393	0.9383	0.9300	0.9100	0.9425	0.9432
